# Usability of an artificially intelligence-powered triage platform for adult ophthalmic emergencies: a mixed methods study

**DOI:** 10.1038/s41598-023-49213-y

**Published:** 2023-12-15

**Authors:** Anish Jindal, Dayyanah Sumodhee, Camilo Brandao-de-Resende, Mariane Melo, Yan Ning Neo, Elsa Lee, Alexander C. Day

**Affiliations:** 1 Moorfields Eye Hospital NHS Foundation Trust, London, UK; 2 Department of Brain Sciences, Institute of Ophthalmology, University College London, London, UK; 3 NIHR Moorfields Clinical Research Facility, Moorfields Eye Hospital, London, UK

**Keywords:** Eye abnormalities, Software, Health occupations

## Abstract

There is growing demand for emergency-based eyecare services where the majority of those attending do not require urgent ophthalmic management. The Royal College of Ophthalmologists have recommended upskilling and supporting of allied health professionals to support eyecare delivery, where machine learning algorithms could help. A mixed methods study was conducted to evaluate the usability of an artificial intelligence (AI) powered online triage platform for ophthalmology. The interface, usability, safety and acceptability were investigated using a Think Aloud interview and usability questionnaires. Twenty participants who actively examine patients in ophthalmic triage within a tertiary eye centre or primary care setting completed the interview and questionnaires. 90% or more of participants found the platform easy to use, reflected their triage process and were able to interpret the triage outcome, 85% found it safe to use and 95% felt the processing time was fast. A quarter of clinicians reported that they have experienced some uncertainty when triaging in their career and were unsure of using AI, after this study 95% of clinicians were willing to use the platform in their clinical workflow. This study showed the platform interface was acceptable and usable for clinicians actively working in ophthalmic emergency triage.

## Introduction

In the UK, ophthalmology is currently the busiest outpatient speciality within the NHS accounting for almost 8 million attendances in 2021/22^1^. In the hospital eye service itself, there is growing demand for emergency-based eye services^2–4^.

With the wide variation in the severity of conditions seen and the high attendance in ophthalmic casualty units, most units have introduced a triage system that allocates a patient to the appropriate category of urgency^5^. It has been found that up to 80% of those attending eye casualty (EC) do not require urgent ophthalmic management following triage and up to 60% were seen and discharged at their first visit^2,6–8^. The Royal College of Ophthalmologists have recommended upskilling and supporting of allied health professionals (AHP) in order to meet this increasing demand for current eye service, where this will enable safe and appropriate management of patients^9^.

Machine learning algorithms could support AHP on improving their accuracy and speed of the triage process. Machine learning algorithms used in the general emergency department have shown that they are better than clinicians at predicting the need for admission^10,11^.

Adoption of medical devices for observation and treatment of patients is becoming more common. User error caused by inadequate medical device usability have become an increasing cause for concern. Many medical devices developed without applying a usability engineering process have found their device to be difficult to learn and impractical by users and may limit its feasibility for use in future studies and clinical practice^12^.

A novel online platform interface with an integrated machine learning algorithm known as the DemDx Ophthalmology Triage System (DOTS) has recently been developed. DOTS has been designed to guide and support AHP clinical decision-making when triaging patients who present in an ophthalmic emergency-based setting.

As far as the authors are aware, there are no studies that have evaluated the usability of any potential artificial intelligence (AI) powered triage platform for ophthalmology. The objective of this study was to evaluate its interface, usability, safety and gain insight on the acceptability of DOTS by AHP before clinical implementation.

## Materials and methods

### Platform development

**Figure 1:**
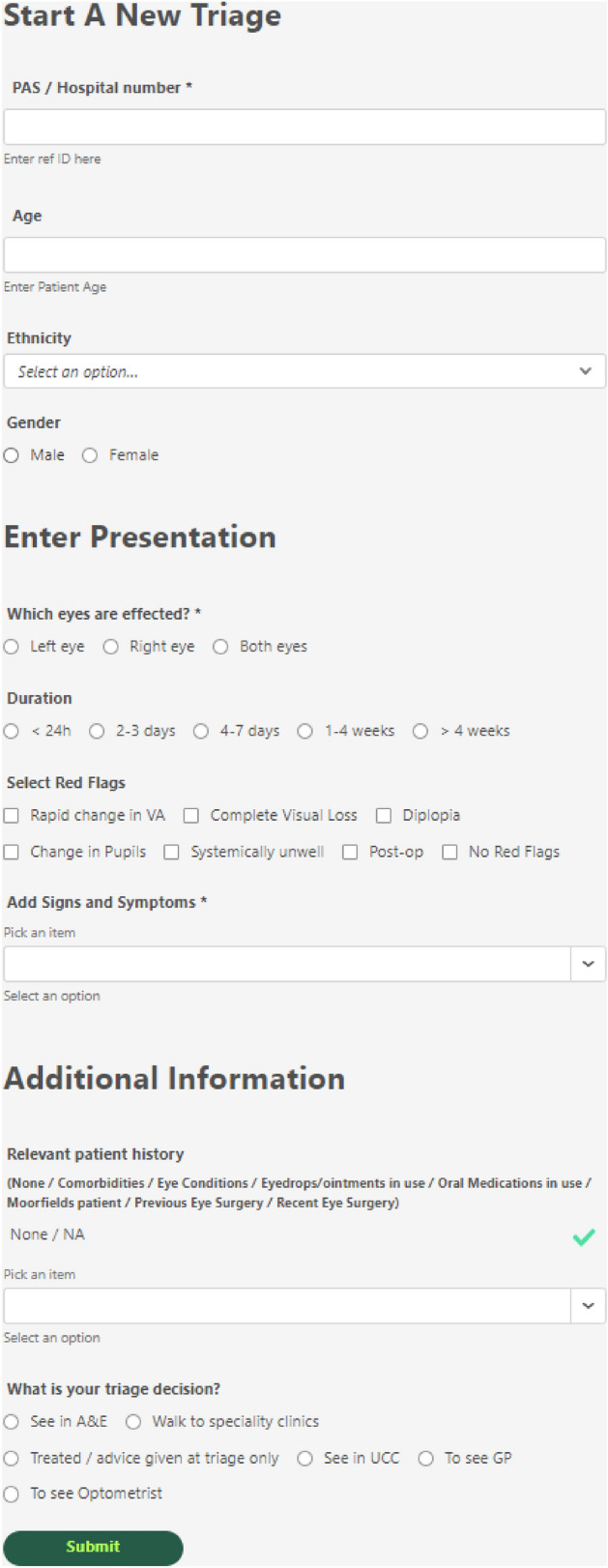
DOTS data entry form for triage clinicians.

The data input form for DOTS (Fig. 1) was developed using the triage form used in Accident and Emergency (A&E) for adults at Moorfields Eye Hospital (MEH) that reflected demographics, presentation, red flags and triage outcomes. The development of ophthalmic signs, symptoms and relevant patient history to select by users were developed using clinical guidelines^13–15^ and by experienced MEH clinicians working in eye casualty. Data was then captured from 12,584 attendances at MEH A&E from 11,733 patients by trained ophthalmic nurses working in triage from 9th August 2021 to April 30th 2022. UK ophthalmic nurses are healthcare practitioners who have a Batchelor’s degree or degree apprenticeship in nursing. They are registered with the Nursing and Midwifery Council and attain ophthalmic subspecialisation through exposure to patients under supervision and completion of competency-based signoffs through internal protocols within their workplace that enable them to work in their designated role. Ophthalmic nurses also have to demonstrate continuing professional development in their field to maintain registration.

**Figure 2:**
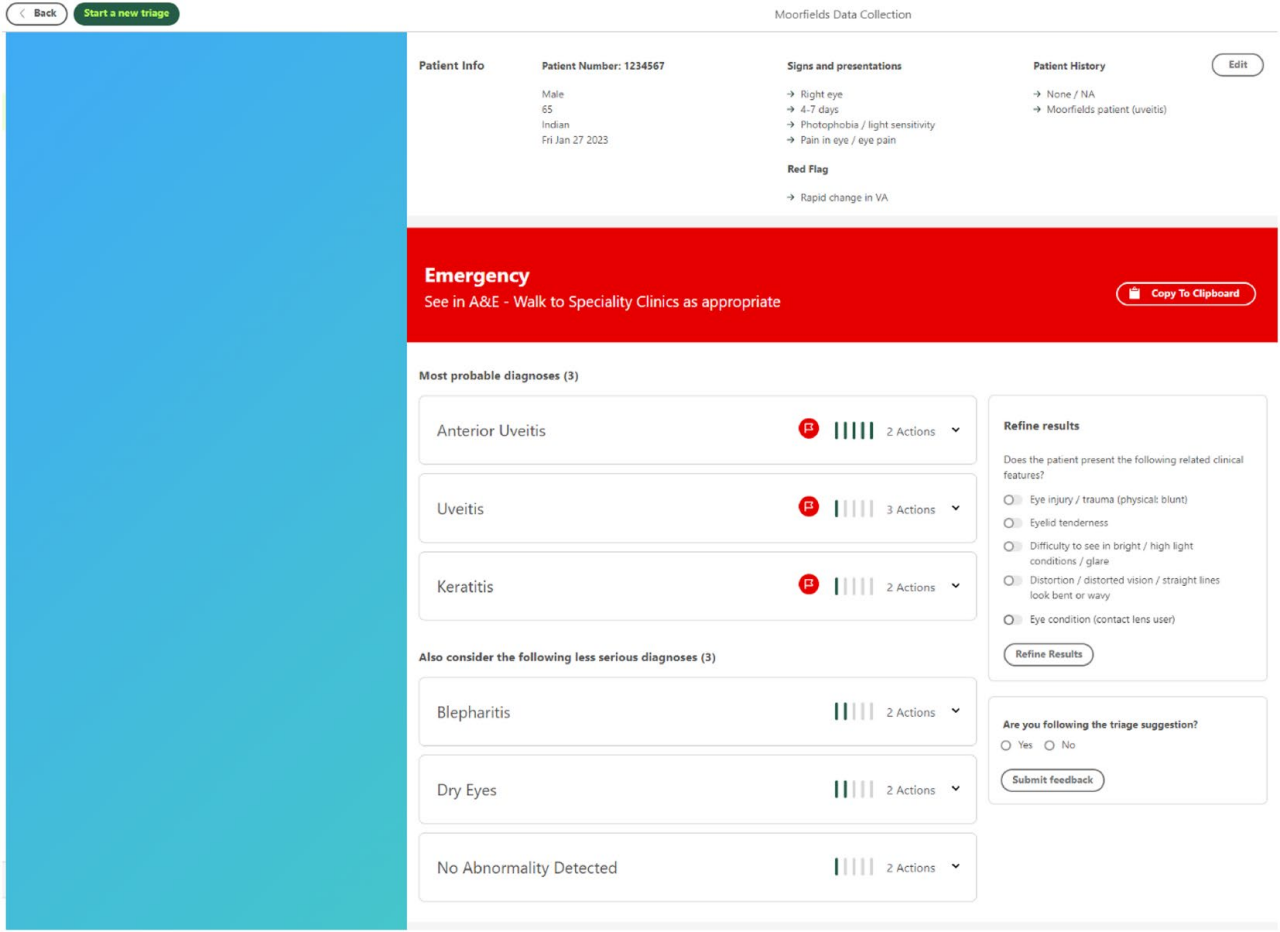
DOTS results page.

The AI algorithm was developed using Python 3.8.10.13 (https://www.python.org) and was trained on the captured data. Four architectures were evaluated for each model, chosen to include simple models as baseline (Logistic Regression and Decision Tree) as well as the ones considered the state-of-the-art for tabular data, including tree bagging (Random Forest) and tree boosting (XGBoost). The model with the highest weighted average of specificities for emergency and urgency was selected as best-performing and was tested using internal and external data sets (1,269 and 761 patients, respectively). This model showed higher specificity and similar sensitivity regarding triage outcomes when compared to ophthalmic nurses, which was then utilised for this study; further details of the development of the algorithm and its’ testing has been described by Brandao-de-Resende et al.^16^. After the user has inputted the data into DOTS, the results page (Fig. 2) displayed the AI suggested triage outcome within a coloured background using a traffic light system that reflects urgency; red: see in A&E same day, walk to specialty clinics same day; yellow: referred to urgent care clinic within the week; green; treated/advice given at triage or see general practitioner/optometrist within a fortnight. For diagnosis prediction, two models were built: (1) the most probable emergency/urgency differentials, and (2) the most probable elective differentials. When the triage model predicts the case as emergency/urgency, a list of the most probable emergency/urgency differentials as “most probable diagnoses” and the list of the most probable elective differentials as ‘consider less serious diagnoses’ were displayed. When the triage model predicts the case as elective, a list of the most probable emergency/urgency differentials as “serious diagnoses to be considered” and the list of the most probable elective differentials as “most probable diagnoses” was shown to the user. With each list of three diagnoses, adjacent probability bars indicating the likelihood of each diagnosis and where appropriate, a red flag indicating the condition would require same day attendance in ophthalmic A&E was displayed. A refine results feature was also incorporated to support history taking and data entry that was based on the algorithm outputs, which could refine the triage and diagnosis as well as a copy to clipboard feature that would enable users to transfer the output for the patient and/or practitioner documentation. Both layout of DOTS, interface and features were developed iteratively based on suggestions and feedback from a multi-disciplinary team of experts and users that included; clinicians working in ophthalmic A&E, an independent research steering committee (comprised of academics and clinicians), patient public involvement panel, a user interface experience designer and digital support from informatics and developers involved in electronic patient records for ophthalmology (https://openeyes.apperta.org) and a Digital Health Accelerator company.

### Usability testing

A prospective mixed methods cohort usability study for the newly developed platform was conducted at MEH NHS Foundation Trust, City Road, UK between October and December 2022.

### Participating clinicians

UK registered ophthalmic professionals based at MEH, City Road, UK were recruited for the study via an email invitation to employed ophthalmic nurses, optometrists and ophthalmologists for their expressions of interest in evaluating the usability of a new artificial intelligence integrated triage platform for use in an ocular emergency setting. Inclusion criteria included qualified clinicians (doctors, nurses, optometrists, other AHP) who actively triage patients who present with ocular emergencies within primary care or an eye casualty setting. Written and informed consent was obtained for all participants, all personal information was pseudonymised and a study number was generated.

The study was approved by the MEH Research Ethics Committee and complied with the tenets of the Declaration of Helsinki. IRAS number 290843. ethics approval number 21/LO/0294, approved on the 4th August 2022.

After the participant had consented, an initial online survey was completed asking information on professional status, gender, days worked in primary/secondary care, years of registration, work experience in eye casualty and experience with digital clinical applications and thoughts on the use of AI to support triage (Supplementary 1).

### Think aloud interview

On completion of the initial survey, participants were booked into an individual single 1-h appointment slot with the researcher either face to face or virtually to test and provide feedback regarding the online triage platform. Participants were provided access to the online platform and were provided instructions on the processes involved at the interview prior commencement. The first stage involved participants interfacing with the platform using an ocular emergency case based on their own experience and ‘thinking aloud.’

The ‘thinking aloud’ is a method used to gather data in usability testing in product design and development, in psychology and a range of social sciences (e.g., reading, writing, translation research, decision making, and process tracing). Think aloud interviews' are a type of qualitative interview where you ask participants to look at/use/engage with the intervention and to say out loud any thoughts that come to mind as they work through it. From a usability testing context, observers can take notes of what participants say and do, without attempting to interpret or influence their actions and words, and especially noting places where they encounter difficulty during the interview as this would represent real-world interaction.

Participants were asked to think aloud regarding the usability and features of the newly developed platform for the data input page and the results page. The researcher observed each participant and followed the method described above. No participant had any prior exposure or usage of the platform. The interview was recorded using audio only via MSTeams and transcripts were autogenerated for subsequent analysis.

### Questionnaire and interview

After the think aloud interview, participants completed an online questionnaire on the presentation, usability, safety, navigation and accessibility of the platform that was divided into 3 sections; patient information, results and overall impression. Answers were rated using either a 5-point Likert scale i.e. 5 as very easy, 1 very difficult) or binary choice (Yes or No). There was also an additional free text boxes where users could elaborate on their feedback about the platform (Supplementary 2).

After the questionnaire, the interviewer conducted a semi-structured interview where participants were asked questions to do an in-depth exploration of their feedback on the platform that was recorded. the researcher expanded on the topics that the participant has described during their Think Aloud session as well as asking them open-ended questions about their platform experience that included what they liked, improvements, how easy or difficult they found navigating the platform challenges and features as well as the platforms’ potential and how likely they were to use it. Before recruitment the study was piloted to refine the interview questions and think aloud task. No data was used from the pilot in the analysis.

### Sample size

Recommendations by the US Food and Drugs Administration for the development of medical devices recommend utilisation of a multidisciplinary team of at least 15 users^17^.

### Statistical analysis

The data captured from the questionnaires were analysed using SPSS Statistics software version 29.0 (http://www.ibm.com/SPSS_statistics). Interview transcripts were analysed using framework analysis, applying the code tree model by Van Waes^18^. This method of analysis starts deductively with a priori codes from the study’s aims and objectives and the subsequent analysis is inductive^19^. DS read and reread the transcripts for familiarisation and data immersion. Transcripts were then coded to create a coding/thematic framework based on the code tree model to identify themes.

The mapping was discussed to ensure codes that could be interpreted as being more than one function were allocated to the most appropriate code tree domain.

**Figure 3:**
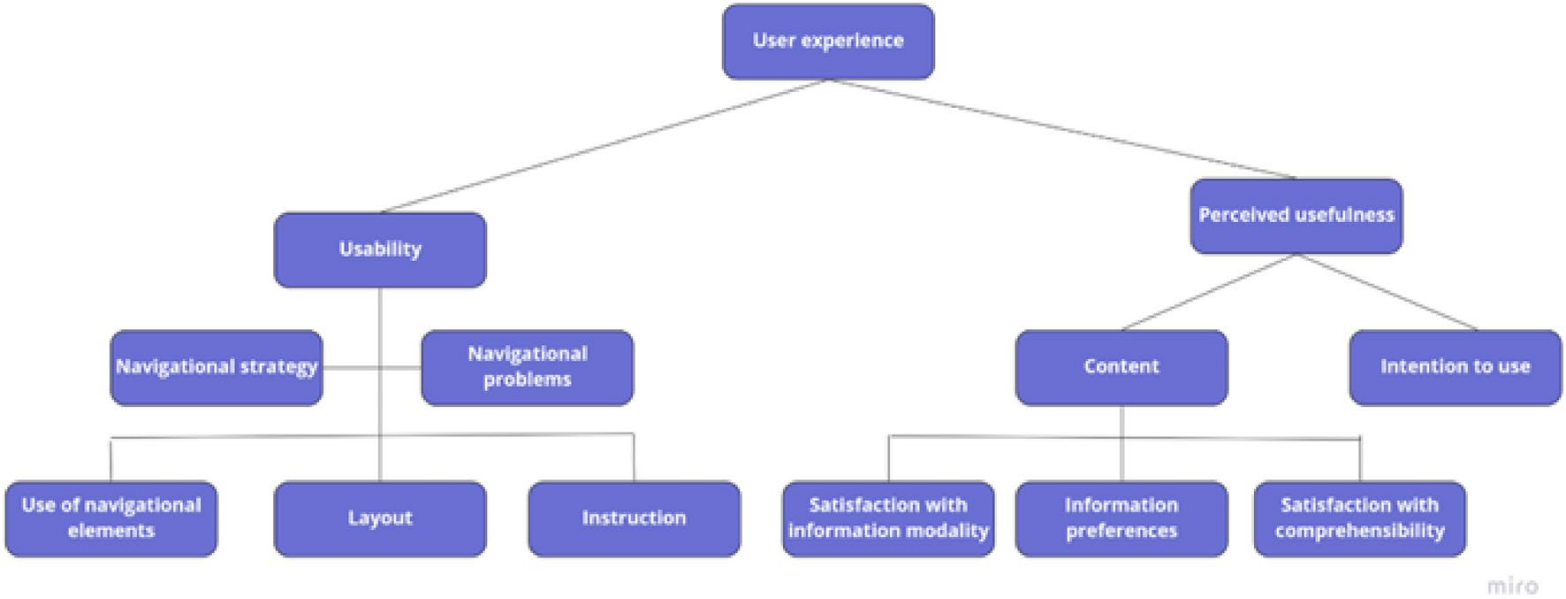
Code tree used for the qualitative analysis.

The code tree analysis model followed two main dimensions: usability and perceived usefulness. The usability was coded from different perspectives as suggested by Van Waes^18^: (1) navigation strategy (i.e., which navigation platforms did the participant use and (2) navigation problems, what were the navigation barriers the participant came across). Three categories were subsequently identified related to the navigation strategies and problems: (1) use of navigational elements, (2) layout, and (3) instructions. Regarding the perceived usefulness of the platform, the negative and positive remarks regarding the content presented on the website with three subcodes: (1) satisfaction with information modality, (2) information preferences, and (3) satisfaction with comprehensibility. Finally, regarding the intention to use the platform were coded in terms of whether and why participants indicated they would or would not use it in the ED setting. The code tree is presented in Fig. 3.

## Results

### Participant demographics

Twenty study participants were included in the study. There were 8 ophthalmic nurses, 7 optometrists and 5 consultant ophthalmologists recruited. The majority of participants (85%) worked in a dedicated eye casualty environment or urgent care clinic in the hospital, further details of the study participants are in Table 1.

**Table 1:** Work experience of study participants.

	No. (%)
Gender (female)	13 (65%)
Years qualified Median (IQR)	22 (17–29)
Work in EC/UCC	17 (85%)
Years worked in EC/UCC Median (IQR)	10.5 (4.3–20.5)
Sessions worked per week in EC Median (IQR)	5.5 (3–9.25)

EC: Eye Casualty; UCC, Urgent Care Clinic.

### Quantitative results

#### Participants experience of digital applications, triage and artificial intelligence

In the initial questionnaire prior DOTS interaction, eight of the participants reported they have used a digital application as a clinical reference guide, where they referred to ease of access and clear instructions as a positive feature for its utilisation and regular use but none had used a digital application to support their clinical decision making. From the 17 participants that have experience in ophthalmic triage in EC, 6 (30%) reported concerns in their current triage process, where a quarter of all respondents specifically mentioned they have experienced uncertainty in triage and had to ‘sometimes seek medical advice’ or ‘refer to protocols.’ 15 (75%) participants were positively receptive to using AI to support triage decisions, four were not sure and one was cautious of triage decisions being dependent on patient verbal input.

### DOTS usability: quantitative

In the subsequent questionnaire that surveyed the participants experience with DOTS revealed that they all found the colours and text were suitable and legible, and they could identify all the information that was inputted before submitting for AI analysis. 90% or more of participants found; they could identify all the input fields; satisfacation with the number of options available for data input selection regarding signs and symptoms; platform order reflected their current triage workflow and were confident in entering the data into all the fields. 10 participants suggested that the signs and symptoms section could be expanded; three participants mentioned one additional symptom each; two participants reported missing symptoms but were already present in the platform; two participants said there should be more symptoms but did not specify which ones; two suggested quantification of symptoms and one suggested free text in the data input.

95% of participants felt the processing time for the algorithm to present an output to be fast, 90% of participants found it was easy to identify the suggested triage output from DOTS and it was clear that the tool’s suggestions were to aid their clinical decision making. 65% of participants did not identify the edit function that could change the inputted data if required. 85% of participants identified a suggested diagnosis was a red flag and they could identify the patient details. 80% were able to interpret the probability bars that were displayed alongside the diagnosis.

Nineteen participants found the triage outcome matched their individual simulated ocular emergency case and one didn’t report this. Ten participants found the diagnosis outcome matched their cases; 5 didn’t discuss this, 4 didn't match and 1 was not sure.

Overall, 85% of participants felt the platform was safe to use in managing patients and the summary report that was generated was acceptable. 90% or more of participants found the platform was easy to navigate through the different sections and easy to use, in addition nearly all participants (95%) were willing to use this tool in their clinical workflow.

### DOTS usability: qualitative

#### Navigational strategies and problems

We identified three categories of navigation strategies that led to problems in optimally navigating DOTS: (1) use of navigational elements, (2) layout, and (3) instructions. Participants frequently commented that it was easy for them to navigate through the different sections of the platform. However, some participants noted navigational issues.

##### Use of navigational elements on data entry and results pages

Most participants commented that the platform was easy to use, and they could easily identify the navigational icons and understand their purpose. Ophthalmologists found the data entry process straightforward.

One suggestion was made to improve the navigational experience related to the addition of a progress bar so users could see their progress to complete the triage, which could be particularly pertinent in an EC setting: “*It would be kind of good if it gives you an idea that ‘don't worry, you don't have another 10 pages to go’*" (Participant 20, consultant ophthalmologist).

Regarding the ‘Edit’ function in the patient’s medical information box on the results page, participants noted that it would be useful to be able to close the box with *a “cross added at the top”* as well as *“greying the ‘refine results’ button at the bottom of the box when no further information has been to be entered”* (Participant 20, consultant ophthalmologist) would improve the navigation flow through the page.

##### Layout of the results page

Some participants commented that more space should be made to contrast the triage outcome with the report of the most probable diagnosis: “*Maybe there should be a couple of spaces in between so that it differentiates*” (Participant 18, nurse).

Most participants appreciated the intuitive and logical layout of the platform, though some did not notice the ‘Edit’ function at the top of the page. In addition, participants found the presentation of the traffic colours (red, yellow, green) useful to highlight the emergency of the triage outcome:

*“It's a good way of doing it because there's something highlighted in red and it’s immediately grabbing your attention. If it's green, you got something that's nationwide. If it’s red, it is an important message.” (Participant 10, optometrist).*

Despite finding the layout of the platform clear, participants acknowledged that receiving further training prior would help to familiarise themselves with the platform more easily: *“I think that once you're taught what the sections are and the purpose of them, then it would become quite easy to use*” (Participant 12, optometrist).

##### Instructions for data entry and results interpretation

Participants were provided with limited instructions prior to the task in order to test the intuitiveness of the platform. Participants appreciated that it was a simple tool to use, nevertheless, they wanted further instructions on how to navigate through the platform.

Two participants highlighted that the summarised instructions needed to be displayed in the data inputting stage without referring to the user guide tab. For instance, information could be added on the first page explaining the data collection process, the different options on the results page including, how to use the ‘Refine results’ tool and copying to clipboard.

#### Perceived usefulness

Perceived usefulness was measured in terms of satisfaction with the content of DOTS and intention to use it. Regarding satisfaction with the content, we identified three categories: (1) satisfaction with information modality, (2) information preferences, and (3) satisfaction with information comprehensibility.

##### Content on the data entry and results pages

*Satisfaction with information modality* Regarding the modality of how information was presented on both the data entry and results pages, participants appreciated the speed of the outcome delivery and the simplicity of the display of the triage outcome: “*It was quick, it didn’t take me to 60 million different screens to get to an answer*” (Participant 10, optometrist). In addition, all participants acknowledged the effectiveness of the implemented strategies in reducing input errors, such as the utilisation of a validation entry for patients’ hospital number, the copy and paste function, and the edit function.

Participants saw the red flags on the results page as a clear way of catching their attention on the importance of the information. However, several participants commented that because of this, the triage decision should be displayed in a clearer way, so it is not overstepped by the diagnosis section, which is a less prioritised outcome from the triage platform:

“*My only concern is the red flag confusion [in the diagnosis section].I think what you're alluding to here is the triage output which has come up because it's a triage platform. It needs to be made a lot bigger, bolder and clearer that this is the [triage] outcome*” (Participant 13, nurse).

The probability bars displayed in the results output were intended to demonstrate the likelihood of a particular diagnosis. Nevertheless, many found them confusing and were unable to interpret the information that the bars represented. This issue is further discussed in the theme of 'Satisfaction with comprehensibility’'.

##### Information preferences

 Participants were satisfied with the triage decisions and the level of information on the platform on both pages. They found the refine results prompts particularly useful as “*this would tease out some other questions you may not have asked the patient and it would help you to add to it”* (Participant 13, nurse) or *"if you've forgotten to ask the question” (Participant 16, nurse).*

In the data entry page, participants often stated that information related to demographics was comprehensive but needed to be more specific about gender and be defined as ‘gender at birth’. Furthermore, they often mentioned preferring entering patients’ date of birth rather than an age since that is what is entered in patients’ medical records. Most participants commented they were satisfied with the number of options when inputting signs and symptoms, however a participant mentioned the significance of asymptomatic patients that was missing from the platform.

*“Yes, we have a lot of patients who come to EC who are asymptomatic and then in letter sent by the opticians, it says they need to be seen in EC”* (Participant 5, nurse).

Participants noted “pain” was missing in the ‘red flag’ checkbox section and in general the platform’s red flags should be expanded to allow adding more red flags options:

*“The triage people need a few more options for red flags. Because I know red eye is red eye but that is actually a red flag as well because it could be a post operation red eye, so I think it has to reflect the ophthalmology side of things as well a bit more”* (Participant 3, consultant ophthalmologist).

##### Satisfaction with comprehensibility of the results

 Participants generally indicated that the red flags and probability bars were useful. However, many were uncertain about interpreting the bars and found it challenging to understand the diagnosis results due to the red flags. Hence, clearer instructions on how to interpret the red flags and probability bars were needed:

“*It wasn't easy to understand because there's so much information. There are two sets of information, as in the these [the ones with red flags] are the ones to consider, and these [without red flags].”* (Participant 4, optometrist)

“*Not sure these bars mean, maybe that is the likelihood?*” (Participant 19, consultant ophthalmologist).

##### Intention to use

Most participants felt that DOTS is a valuable triage platform and intended to use it in the future, although consultant ophthalmologists were less likely to find the platform useful for themselves than other healthcare professionals potentially due to their extensive training and experience working in the EC setting.

Participants often cited the platform as particularly helpful to AHP or those who are in training or are less experienced in acute ophthalmology and can support their decision making in triage:

“*This is a very good platform for healthcare professionals. The area of triage is a bit alarming for some of us, who are new and having a backup system of this nature helps. It will be a supportive tool for whoever's coming to do triage so they are not alone in that room trying to make decisions all by themselves.*” (Participant 13, nurse).

Practitioners found the 'most probable diagnoses' feature helpful. They viewed it as an additional source of information and support for clinical decision making or referring patients in the eye care pathway:

“*Well, it [the most probable diagnosis feature] will help in terms of shortening, the time that they're in charge, and you'll be able to decide if the patients need to stay in or they could be redirected somewhere else straight away in terms of the referral*.” (Participant 20, nurse).

## Discussion

Device safety has been found to be correlated to the usability of a product^20,21^, where usability has been identified as a key component of good practice in the development of digital applications^22^. In this study, most clinicians found the tool safe to use, easy to navigate, rapid in providing an output and were willing to use the platform in their workflow that would support these themes for clinical integration. A quarter of clinicians had reported feeling uncertainty when undertaking ophthalmic triage in their career, which was surprising considering most were experienced clinicians where four out the five had least 18 years of clinical experience and it is often the assumption that those with more experience, would have greater confidence in clinical decision making^23^. With the DOTS tool having high concordance with the participants expected triage outcomes (95%) and the positive reception regarding users being able to refine the symptoms, this may have impacted clinician’s acceptability and perception of patient safety; where there was an increase of twenty percent willingness to use an AI assisted tool pre and post study. These findings are supportive of other studies that have found clinicians’ acceptance and attitudes towards electronic health record systems are related closely to system usability^24–26^.

Putting user experience in the context of Web-based health information tools, evaluation of usability and perceived usefulness in terms of content and intention to use the tool is important^27^. Where usability is related to the ease of use of a system, perceived usefulness addresses “the degree to which a person believes that using a particular system would enhance his or her job performance”^28^. These themes were explored in the think-aloud section of the study, where participants found that the data fields were comprehensive with the red flags and probability bars providing useful information. However, there was uncertainty in the interpretation of the latter by twenty percent of the cohort and a third of participants missed the edit function within the platform. Whilst participants were provided with instructions prior commencement of the study with an explanation regarding the platform’s features, this could have led to information overload as this was the first time they had interacted with the platform that may have resulted in misidentification and misinterpretation for some users. With medical devices being an integral part of clinical practice in healthcare settings, nurses are facing an increase in the number of these with varying degrees of complexity^29,30^, it’s therefore vital that the provision of both initial and ongoing training on device management and use complement good device designs to foster positive attitudes^31^, which can also minimise the risk of misinterpretation. Other aspects of usability are the extent to which the tool meets the user’s needs and abilities in terms of navigation strategy and problems^18^. Navigation was acceptable in DOTS where participants appreciated the intuitive and logical layout of the platform that reflected their triage workflow and use of the traffic light system to guide interpretation. However, some of the navigational problems were attributable to the layout of the platform, for instance, the ability to close the refine results box and the need for more space between the triage outcome and the most probable diagnosis for ease of distinction; as the triage outcome is the primary function of the platform where nearly all the participants found the triage outcome matched their simulated ocular emergency. This re-enforces the iterative process of medical devices that usability studies are conducted to identify any problems that need addressing before clinical use where the number of participants for this study was sufficient^32^.

The US Food and Drug Administration for the development of medical devices recommended a multidisciplinary team approach, with at least an inclusion of 15 users^17^. One of the major strengths of this study was the number of stakeholders across several disciplines that were involved in the development of the triage tool; usability was then evaluated by the relevant number of clinicians who actively work in eye casualty, signifying the applicability of testing for the target users of the device. Taking a user-centred approach to development increases the likelihood of a device being used regularly, correctly and users being satisfied^32^. Think-aloud observations are a classic method to assess user experience of Web-based interfaces^33^, an additional strength of this study was to use this in conjunction with the quantitative survey, where this combination can support validity^34^. Whilst the tool’s usability was evaluated by experienced clinicians working in its intended setting; some clinicians recommended that this tool would be useful for those in training or who were less experienced but these groups were not evaluated, which may limit the generalisability of the study’s findings. Furthermore, this study may have induced a Hawthorne effect for the evaluated healthcare practitioners particularly if their performance is being measured.

In conclusion, the DOTS tool was found to be easy to use, reflected users current data entry at triage and was easy to navigate as well as being fast and matching their expected triage output amongst the majority of clinicians who examine ocular emergencies. Nearly all practitioners indicated that they would use the platform following formal device validation when triaging patients and recognised that the tool’s purpose was to aid their decision-making. The study identified features in the platform that required improvements to the interface to increase usability and the need for further clarification to users, re-enforcing the importance of clear instructions being provided for all its’ functions prior use. After validating the tool in terms of usability, further research is required to evaluate DOTS in a real-world clinical trial that is currently underway to determine its performance and usability with clinicians working in ophthalmic triage with a range of expertise, where continuing feedback will support the iterative process of platform development.

### Supplementary Information

Supplementary Information 1.

Supplementary Information 2.

## Data Availability

The datasets generated during and/or analysed during the current study are available from the corresponding author on reasonable request.
