# Supplementary material for: Usability of an artificially intelligence-powered triage platform for adult ophthalmic emergencies: a mixed methods study
Source: Sci Rep. 2023 Dec 15;13:22490. doi: 10.1038/s41598-023-49213-y (PMC10728059; doi:10.1038/s41598-023-49213-y)
Supplement: Supplementary file 1 — Supplementary Information 1. [file 41598_2023_49213_MOESM1_ESM.docx]

# DemDx Triage Platform Usability Study Part 1

**Introduction**

Dem Dx's Artificial Intelligence-powered clinical reasoning platform is designed to support triage decisions in those attending ophthalmic casualty. The intended users of this device will be healthcare professionals that perform triage in acute clinical settings. Users will receive training on how to operate the software safely and effectively before clinical use.

Users will access the Dem Dx AI-driven Ophthalmology triage platform with their personal login details via a web-based application and start the triage process by entering patient information. This information will then be used by the machine learning algorithm (artificial intelligence) to predict the most likely diagnoses and return to the user with a structured review of the clinical case, triage referral outcome, and the most likely diagnoses with management.

It is expected that initial performance of the developed platform will then be evaluated against experienced ophthalmologists.

We would like your feedback on the usability of the platform

**Date:**

**Study Number:**

**Please can you complete the following questions**

### Section 1: Participant demographics

1. What’s your professional background?

- Optometrist

- Orthoptist

- Nurse

- Technician

- Doctor (non-ophthalmologist)

- Ophthalmologist

- Other: please specify

1. Are you:

- Male (He/His)

- Female (She/her)

- Prefer not to say

1. Do you work in primary care?

If no, please go to question 4

If yes:

1. If yes, How many sessions have you worked in a week in primary care in the last 12 months? (One session is half a day)
2. How many years have you worked in this setting?
3. On average, how many patients do you examine that present with an acute ophthalmic condition in a week?
   1. 0
   2. 1-5
   3. 6-10
   4. 11-15
   5. 16-20
   6. >20
4. Do you work in a hospital-based setting?

If no, please go to question 5.

If yes:

1. How many sessions have you worked in a week in a hospital-based setting in the last 12 months? (One session is half a day)
2. How many years have you worked in this setting?
3. Which clinics are you currently working in?
4. Are you currently working in an eye casualty department or a casualty-based clinic?

-Yes

-No, please go to question 6.

If yes:

1. How many years have you worked in this clinic?
2. How many sessions in a week in the last 12 months? (One session is half a day)
3. On average, how many patients do you examine that present with an acute ophthalmic condition in a week?
   1. 0
   2. 1-5
   3. 6-10
   4. 11-15
   5. 16-20
   6. >20
4. In which year did you initially register with the GOC/GMC/NMC/HCPC, etc?

#### Section 2: Experience with digital clinical systems and expectations

1. Have you ever used a digital application to support your clinical decision or to use it as a clinical reference guide? For example i.e microguide, vision tests, etc
   1. Yes
   2. No

If yes,

1. Which ones have you used and what did you use it/them for?
2. What did you find in the application that made it accessible for you to use?
3. Are you still using the application?

If No, why did you stop using it? Were there things that made it difficult to use?

1. Are there any steps in your current triage workflow that you have any concerns with or are unsure what to do?
2. What are your thoughts on using artificial intelligence to support triage decisions such as provisional diagnosis and management?
