# Supplementary material for: Usability of an artificially intelligence-powered triage platform for adult ophthalmic emergencies: a mixed methods study
Source: Sci Rep. 2023 Dec 15;13:22490. doi: 10.1038/s41598-023-49213-y (PMC10728059; doi:10.1038/s41598-023-49213-y)
Supplement: Supplementary file 2 — Supplementary Information 2. [file 41598_2023_49213_MOESM2_ESM.docx]

# DemDx Triage Platform Usability Study Part 2

**Date:**

**Study Number:**

**Please can you complete the following questions**

**Questionnaire**

Now that you have gone through the platform, please can you complete the following questionnaire. You can go back to the platform for your reference at any time

**Patient information page**

1. Was the text legible?

Yes

No

1. Were the colours suitable?

Yes

No

1. Could you identify all the fields that required your input?

Yes

No

1. To what extent were the input fields ordered in a way reflects your current triage process?
   1. Very similar
   2. Fairly similar
   3. Neutral
   4. Fairly dissimilar
   5. Very dissimilar
2. How satisfied were you with the number of options in the drop-down menus when inputting patient signs and symptoms?
   1. Very satisfied
   2. Fairly satisfied
   3. Neutral
   4. Fairly dissatisfied
   5. Very dissatisfied
3. Were there any particular signs or symptoms you felt were missing of the dropdown menu that were related to your case?

If so, please detail

1. Before proceeding with the submit button, could you identify all the information that has been inputted?

Yes

No

1. How confident were you in inputting the data into all the required fields?
   1. Very confident
   2. Fairly confident
   3. Neutral
   4. Fairly unconfident
   5. Very unconfident
2. How worried were you that you might make input errors?
   1. Very worried
   2. Fairly worried
   3. Neutral
   4. Fairly comfortable
   5. Very comfortable
   6. Don’t know

#### Results page

1. Was the text legible on this page?

Yes

No

1. Please rate the time taken to receive the summary report?
   1. Very fast
   2. Fairly fast
   3. Neutral
   4. Fairly slow
   5. Very slow
2. Please rate your ability to locate patient identifiable details.
   1. Very easy
   2. Fairly easy
   3. Neutral
   4. Fairly difficult
   5. Very difficult
3. How easy or difficult was it to identify the urgency of the referral?
   1. Very easy
   2. Fairly easy
   3. Neutral
   4. Fairly difficult
   5. Very difficult
4. How suitable were the colours that reflected the urgency of referral?
   1. Very suitable
   2. Fairly suitable
   3. Neutral
   4. Fairly unsuitable
   5. Very unsuitable
5. Please rate your ability to identify each of the potential diagnosis and their respective probabilities.
   1. Very easy
   2. Fairly easy
   3. Neutral
   4. Fairly difficult
   5. Very difficult
6. Could you identify the chance of a red flag condition(s)?
   1. Yes
   2. No
7. Did you identify the edit function in the clinical information section that could change the information that was originally inputted?
   1. Very easy
   2. Fairly easy
   3. Neutral
   4. Fairly difficult
   5. Very difficult
8. How clear was it in the platform that the results are only suggestions to aid the user in their decision making?
   1. Very clear
   2. Fairly clear
   3. Neutral
   4. Fairly unclear
   5. Very unclear
   6. Don’t know

#### Overall impression

1. How safe or unsafe do you feel this platform is in the context of managing patients?
   1. Very safe
   2. Fairly safe
   3. Neutral
   4. Fairly unsafe
   5. Very unsafe
2. How did you find it to navigate through different sections of the platform?
   1. Very easy
   2. Fairly easy
   3. Neutral
   4. Fairly difficult
   5. Very difficult
3. How easy or difficult was it to use the platform?
   1. Very easy
   2. Fairly easy
   3. Neutral
   4. Fairly difficult
   5. Very difficult
4. The summary report has a copy to clipboard and pdf function to export the information. How acceptable would you find this platform to use in your clinic workflow?
   1. Very acceptable
   2. Fairly unacceptable
   3. Neutral
   4. Fairly unacceptable
   5. Very unacceptable
   6. Don’t know
5. Would you be willing to use this platform as a part of your clinical workflow?
   1. Very willing
   2. Fairly willingly
   3. Neutral
   4. Fairly unwillingly
   5. Very unwillingly
